# Depression with comorbid juvenile-onset fibromyalgia syndrome novel treatment plan using interpersonal psychotherapy: A case report

**DOI:** 10.1097/MD.0000000000039505

**Published:** 2024-09-06

**Authors:** Samah H. Alkhawashki, Norah Algarzae

**Affiliations:** a Department of Psychiatry, College of Medicine, King Saud University, Riyadh, Saudi Arabia; b Department of Physiology, College of Medicine, King Saud University, Riyadh, Saudi Arabia.

**Keywords:** adolescents, comorbidities, depression, duloxetine, fibromyalgia syndrome, interpersonal psychotherapy, juvenile-onset fibromyalgia

## Abstract

**Background:**

Fibromyalgia syndrome (FMS) affects 2% to 4% of people, with increasing prevalence in Saudi Arabia reaching 13.4%. FMS can occur in adolescents, known as juvenile-onset fibromyalgia (JFM) with comorbidities including depression, anxiety, and psychological stress. Our patient presented to the child and adolescent psychiatry clinic at King Saud University Hospital Medical City. A year before coming to our clinic, at the age of 15 she was initially diagnosed with JFM followed by a comorbid persistent depressive disorder.

**Methods:**

As a novel treatment method, a combination treatment approach was used, including a pharmacological intervention with Duloxetine, and a non-pharmacological intervention with interpersonal psychotherapy for adolescents. She completed 16 weeks of therapy while monitoring for duloxetine response and side effects.

**Results:**

Depressive symptoms were in remission by treatment’s end and continued to be in her first month posttreatment follow-up, and the FMS symptoms were also controlled.

**Conclusion:**

Our present case highlights a combined approach to treat depression and JFM in adolescents as a novel intervention method thus we strongly recommend utilizing it for similar cases.

## 1. Introduction

Fibromyalgia syndrome (FMS) is a chronic debilitating syndrome that affects 2% to -4% of people.^[1]^ In FMS, there is a lack of primary tissue damage with evident neuropathic pain described by patients as dull/deep/aching or burning/tingling with varying locations and levels of intensity.^[2,3]^ Juvenile-onset fibromyalgia (JFM) is a form of FMS that is a complicated chronic disease that remains hard to understand in adolescence. FMS comorbidities include cognitive functions and psychological stressors such as anxiety and depression.^[4,5]^ FMS is described as masked depression.^[6]^ Depression occurs in FMS patients 40% to 80% of the time,^[6]^ due to the shared symptoms and pathophysiology.^[7]^ Similar physiological pathways in FMS and depression are due to the commonality in the initiation of the acute stress response. Once activated, the acute stress response activates the hypothalamic-pituitary-adrenal (HPA) axis.^[7]^ In addition to activating the HPA axis, pain pathways from FMS and depression can also activate the autonomic nervous system (ANS).^[2]^ In FMS, the activation of the HPA axis and ANS causes the release of corticotrophin-releasing hormone causing the release of cortisol thus initiating a reaction with the neurotransmitter system and activating the “fight-or-flight” response.^[2,7]^ Similarly, in depression, the activation of the HPA axis leads to an increased level of circulating inflammatory cytokines, further contributing to depression progression by decreasing serotonin levels.^[8]^ Globally, the activation of the HPA axis is linked to adulthood onset of depression; however, there is less information on its mechanism of action in adolescents.^[9]^ A systemic review and meta-analysis show that raised levels of cortisol due to the HPA axis in adolescents is a risk factor for depression.^[8]^ The average age of adolescents diagnosed with JFM is between 13 and 15, depending on socio-cultural background.^[10]^ JFM has yet to be fully understood, specifically how chronic pain, and social, and physical distress are life-altering for adolescents.^[11]^ The likelihood of depression incidences in the beginning stages of life increases as time goes on, we have a perfect window for targeting JFM and depression for new methods of treatment.^[8]^

## 2. Case presentation

### 2.1. Patient description

Our patient is a 15-year-old female living with her parents and younger sibling in Riyadh, Saudi Arabia. She lived abroad during her formative years while her parents continued their higher education. When she was 8 years old, the family moved to a city in Saudi Eastern Providence and lived there for 2 years. Currently, she is 15 years old and studying at an international school. Her father described her as smart, calm, and patient. She presented to the child and adolescent psychiatry clinic at King Saud University Hospital Medical City, with her father, due to depressive symptoms, as a referral from the rheumatology clinic. A year before coming to our clinic, she was diagnosed with JFM; during which she was given recommendations to exercise, stretch, and use paracetamol for pain episodes.

### 2.2. Case history

First, regarding our patient’s pain, she reported her pain was in her lower extremities starting at 4 years old; as she grew older the pain started to generalize, especially around the age of 10 and it reached its peak last year (age 14). She underwent a thorough investigation by neurology and rheumatology and was diagnosed with JFM. Her pain has been disturbing her sleep and it is generalized and felt in her legs, arms, feet, hands, back, and shoulders occurring daily and worsening with stress. She copes with it by lying down, taking paracetamol-based painkillers, and wrapping her extremities with warm compressors, which she reported do not always help. The pain has affected her social and academic functioning immensely. She has been trying to engage in cardio exercises, however, she often feels tired and cannot complete the workouts.

Second, in terms of her mood, our patient reported that she started noticing symptoms of low mood when she moved to Riyadh at the age of 10; during this time, she had to deal with the transition to a new school where she was bullied and excluded by the students. At 12 years old, the pain started to worsen, she had increased appetite, initial and middle insomnia, decreased concentration, energy slowness, a sense of hopelessness/worthlessness, and guilt feelings towards her parents about burdening them with her illness. Her persistent pain and continuous bullying were her stress factors then. The low mood and loss of interest became more apparent, reaching its peak when she was 13 years old. Her mental health state continued to decline, and at age 14, she started having passive suicidal ideas however she had no active suicidal ideas, intent or plan, negatively affecting her relationships with family and friends due to isolation and withdrawal. The presented stress factors affected her academic functioning and ability to excel at school despite her continued effort. Two months prior to her JFM diagnosis, the depressive symptoms moderately improved, possibly due to having low stress over the summer vacation or having the Holy Quran verses read to her by a spiritual healer, or due to the excitement of knowing that she is starting in a new school away from bullies.

Third, regarding her review of symptoms, no anxiety symptoms were identified, nor were manic symptoms, psychotic symptoms or substance use. The patient denied a history of emotional, physical, or sexual abuse. As for development and early childhood, her mother’s pregnancy was normal; no history of smoking, alcohol, or illicit drug usage during pregnancy. Our patient was born through normal vaginal delivery at full term. During her formative developmental years, she timely met milestones. As a child, he was shy, sensitive and calm. She was selective in playing with other children.

Lastly, in terms of her past medical, surgical, psychiatric history, and family history. She has vitamin D deficiency corrected with supplements and iron deficiency anemia corrected with ferrous fumarate. She uses paracetamol for pain episodes. She has no known drug allergies, no history of head trauma, cardiac conditions or seizures, and no history of previous psychiatric or psychological interventions. In terms of family history, no major psychiatric history was reported, however, mood symptoms were reported in first- and second-degree relatives. Additionally, there is a family history of rheumatological conditions including rheumatoid arthritis and juvenile arthritis.

In the past our patient has had multiple challenging transitions in her life altering her relationships and social functioning skills; as she described 1 of the biggest transitions was moving to Saudi Arabia after living abroad from birth to 8 years old. This transition was hard on her due to the language barrier, and due to her father staying behind for 2 years to finalize his studies aboard. The patient describes feeling that her parents hold her to high expectations, so this constantly leads her to feel like she is a disappointment to them. She has a fair relationship with her sibling. As for friendships, the patient reported that she does not have friends or romantic partners. In terms of her school performance, initially, she did not have any challenges at school, however over the past 2 years the pain and mood symptoms made school difficult for her. Before the COVID-19 pandemic, she reported constant bullying and exclusion behavior from her classmates, causing her to lose motivation and focus from online teaching.

### 2.3. Assessment

Based on the assessment done in our clinic, the patient has fulfilled the Diagnostics Statistical Manual-5 criteria for early onset of persistent depressive disorder with intermittent major depressive episodes and current episodes.^[12]^ The patient’s current episode was mild in severity; however, the previous episodes were moderate to severe. Since our patient has JFM, she is at higher risk of developing mood and anxiety disorders.^[13]^ In addition, she has vitamin D deficiency and anemia, both of which affect mood.^[14,15]^ There is a possible family history of mood disorders, which puts her at increased risk of developing it herself. According to a psychosocial perspective, the patient has a sensitive and perfectionistic temperament. Studies have shown that children with perfectionistic temperaments are at increased risk of having high expectations of themselves, and negative self-perceptions when their goals are not reached.^[16,17]^ Based on her social assessment, in our clinic, we found that she has been struggling with relationships both on family and friendship levels which we assessed to perpetuate the depressive symptoms she is experiencing leading to more isolation.

### 2.4. Treatment and outcome

Treatment options were discussed with our patient, and we described the novelty of our combined treatment approach; including pharmacological treatment through Duloxetine and non-pharmacological treatment through interpersonal psychotherapy (IPT). Duloxetine was conferred as a good option for the treatment of both JFM and depression based on adult data.^[18]^ Duloxetine acts as a mixed serotonin and norepinephrine reuptake inhibitor, thus it increases serotonin and norepinephrine levels.^[19]^ After discussing the risks and benefits with our patient, the medication was started at 30 mg orally daily and increased after 1 week to 60 mg orally daily. As for the non-pharmacological interventions, based on the literature psychotherapy options were discussed, IPT for Adolescents using the bidirectional model has shown promising results.^[20]^ IPT has 3 main sessions (beginning, middle, and end).^[21]^ The manual used for treatment is the interpersonal psychotherapy for depressed adolescents (IPT-A) manual.^[22]^ The sessions were planned weekly with the plan to complete a full course of therapy of 14 to 16 week (about 3 and a half months). During treatment, the lab tests were constantly monitored for vitamin D levels and anemia. In addition, the duloxetine response and side effects. Initially, our patient experienced side effects of nausea and headache on the dose of 30 mg orally daily. Hence, we took a slower titration approach than our initial decision and increased the dose a month after initiating the medication, to 60 mg orally daily and maintained it.

IPT Interpersonal therapy is divided into 3 phases, initial, middle, and termination phase.^[23]^ The initial phase was done over 4 sessions. During this phase, we discussed the bidirectional relationship between depression and interpersonal relationships, and we introduced the possibility of pain being a perpetuating factor for depressive symptoms.

We worked on the closeness circle and interpersonal inventory during which we discussed the 4 foci of IPT-A (interpersonal role transitions, interpersonal disputes, grief, and interpersonal deficits). Then we decided on the focus of interpersonal role transitions which was decided considering her transitioning into a youth living with chronic pain syndrome. Using the limited sick role was discussed with the patient and her family, explaining the sick role she has been living with during the depressive phase, and how it was exacerbated by the pain syndrome. We discussed how this sick role needs to be limited by engaging in activities with her family to be able to activate herself past the depressive and pain symptoms. The family understood the plan and agreed to work together during the middle phase of therapy. The parents actively participated in the therapy process and presented at least every 3 sessions for check-ins to discuss our patient’s progress. The middle phase took place over 7 sessions, during which we used different techniques of therapy including communication analysis, role plays, and reviewing new and old roles, which in turn helped to assess and develop social skills to place a support plan for her transition into the new role of being a young lady living with a chronic pain syndrome.

We worked immensely on the ability to request help when she needed it since this was a major deficit that we explored and found was due to the fear of burdening her family with her struggles. In addition, the communication process with the different family members was considered in detail and this helped in developing better communication skills, which was evident by the feedback gathered by the parents.

**Figure 1.**
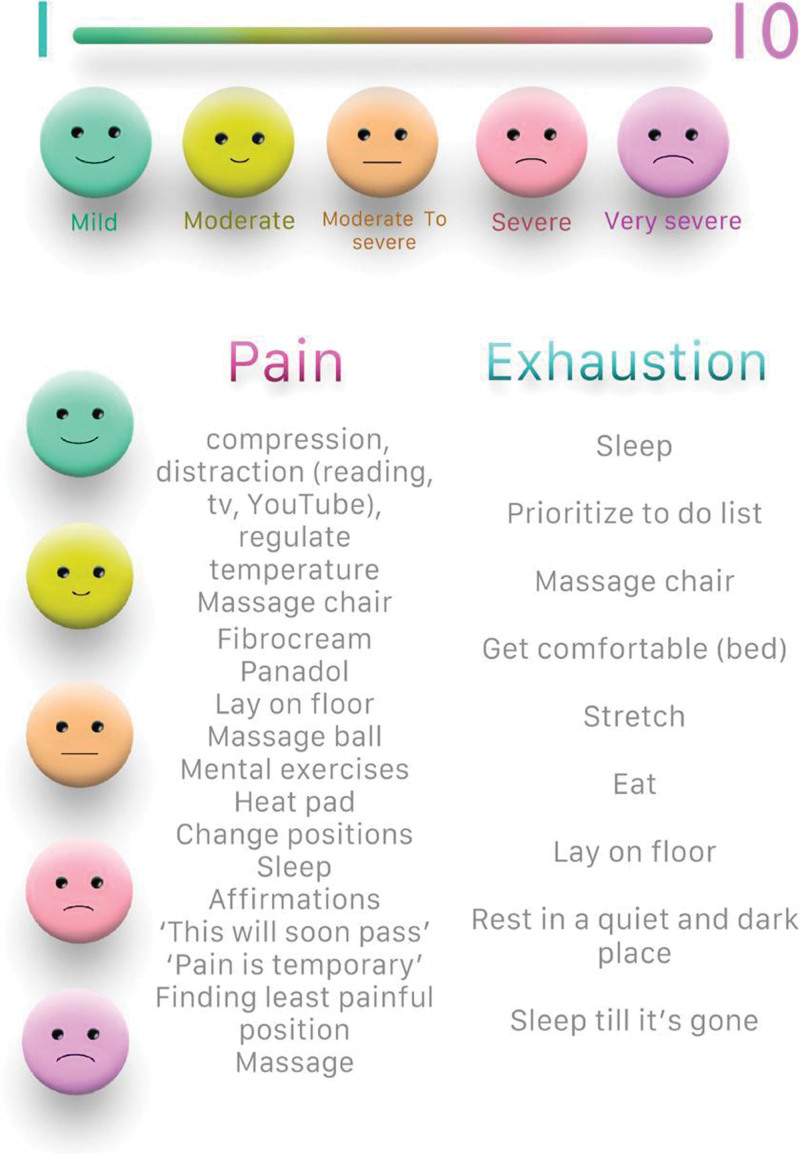
A stepwise approach to manage the pain and exhaustion flare-ups depending on the severity of the symptoms.

Additionally, we came up with a stepwise approach to managing the pain and exhaustion flare-ups depending on the severity of the symptoms (Fig. 1). This approach helped her plan for the episodes and share her needs with her family ahead of time, to minimize any frustration and miscommunication during the episodes.

The termination phase took place over 4 sessions, during which the symptoms shifted to full remission towards the end of the middle phase. In the final phase, we consolidated the skills she had gained during the middle phase and explored the meaning of ending therapy, which included uncertainty found in her feeling of accomplishment and concurrent fear of having to become “her own therapist” in the process. A booster therapy session was provided 1 month after the final IPT-A session during which she reported continued remission of the depressive symptoms and continued to be motivated to use the skills she had learned to manage her mood and pain symptoms. She was complaining of challenges with fatigability, pain, and increased academic stress due to progressing in her academics. Hence, we increased the dose of duloxetine to 90 mg orally daily despite the limited evidence for pain management when increasing the dose past 60 mg. Furthermore, on regular every-3-month follow-ups, she showed well-managed fibromyalgia symptoms with occasional flare-ups related to stress levels. She continues to be maintained on duloxetine 90 mg orally daily. The Beck Depression and Anxiety Inventories^[24]^were done during each phase of treatment and they showed a gradual improvement in symptoms towards the end of therapy, which supported the improvement witnessed clinically.

## 3. Discussion

Depression can commonly occur when patients have chronic medical conditions including muscular diseases.^[25]^ In addition to age, our patient’s diagnosis of JFM puts her at a higher risk of developing depression; studies have linked JFM to an increased risk of mood and anxiety disorders.^[13]^ Exhibiting other underlying biological factors our patient was diagnosed with vitamin D deficiency and anemia, which were controlled by supplements. The literature previously linked vitamin D deficiency and anemia to depression.^[14,15,26]^ Additionally, she presented with an underlying perfectionistic temperament an important psychological factor in her presentation, because it is linked to psychopathology including anxiety and depression in adolescent patients.^[17]^

JFM is a complicated chronic disease that remains hard to understand in adolescence. Considering that our patient presented with a severe level of both JFM and depression we chose a combination treatment approach by combining pharmacological and non-pharmacological treatments. Although the combined treatment method has shown positive outcomes,^[27]^ we are the first to treat JFM and depression through it. Pharmacologically we used 60 mg oral Duloxetine daily to treat pain and depression; clinical trials using the same dosage have previously shown significant positive outcomes on FMJ adult patients.^[18]^ The non-pharmacological method we used is IPT for adolescents, during which our patient had 14 to 16 weeks of therapy. Our patient showed positive results in her first month of posttreatment follow-ups and in all remaining follow-ups. IPT has been shown to positively treat major depression^[28]^ and mood.^[21]^ However, our case is the first to report the importance of combining the duloxetine and IPT and we were successful in reaching a positive outcome.

## 4. Conclusion

JFM comorbidities are widespread persistent pain that prevails in adolescents and may continue into adulthood.^[29]^ In adolescents, the average age of diagnosis of JFM is between 13 to 15, similarly, at that age, there is a higher likelihood of depression incidences.^[8,10]^ We report in this case a 15-year-old patient diagnosed with depression and comorbid JMS. As a novel intervention, we highlight the positive impact of a combined approach to treating both depression and an underlying chronic syndrome JFM in adolescents.

## Author contributions

**Conceptualization:** Samah H. Alkhawashki, Norah Algarzae.

**Data curation:** Samah H. Alkhawashki, Norah Algarzae.

**Formal analysis:** Samah H. Alkhawashki, Norah Algarzae.

**Writing – original draft:** Samah H. Alkhawashki, Norah Algarzae.

**Writing – review & editing:** Samah H. Alkhawashki, Norah Algarzae.
